# Finite element analysis of cutting balloon expansion in a calcified artery model of circular angle 180°: Effects of balloon-to-diameter ratio and number of blades facing calcification on potential calcification fracturing and perforation reduction

**DOI:** 10.1371/journal.pone.0251404

**Published:** 2021-05-13

**Authors:** Xiaodong Zhu, Mitsuo Umezu, Kiyotaka Iwasaki

**Affiliations:** 1 Department of Modern Mechanical Engineering, School of Creative Science and Engineering, Waseda University, Shinjuku, Tokyo, Japan; 2 Department of Integrative Bioscience and Biomedical Engineering, Graduate School of Advanced Science and Engineering, Waseda University, Shinjuku, Tokyo, Japan; 3 Cooperative Major in Advanced Biomedical Sciences, Graduate School of Advanced Science and Engineering, Waseda University, Shinjuku, Tokyo, Japan; National University of Ireland Galway, Galway, Ireland, IRELAND

## Abstract

Calcified artery lesions cause stent under-expansion and increase the risk of in-stent restenosis and stent thrombosis. Cutting balloons facilitate the fracturing of calcification prior to stent implantation, although vessel dissection and perforation are potential issues. In clinical practice, calcifications having maximum calcium angles ≤ 180° are rarely fractured during conventional balloon angioplasty. We hypothesize that the lesion/device diameter ratio and the number of blades facing a non-circular calcified lesion may be crucial for fracturing the calcification while avoiding vessel injury. The geometries of the cutting balloons were constructed and their finite-element models were generated by folding and wrapping the balloon model. Numerical simulations were performed for balloons with five different diameters and two types of blade directions in a 180° calcification model. The calcification expansion ability was distinctly higher when two blades faced the calcification than when one blade did. Moreover, when two blades faced the calcification model, larger maximum principal stresses were generated in the calcification even when using undersized balloons with diameters reduced by 0.25 or 0.5 mm from the reference diameter, when compared with the case where one blade faced the calcified model and a balloon of diameter equal to the reference diameter was used. When two blades faced the calcification, smaller stresses were generated in the artery adjacent to the calcification; further, the maximum stress generated in the artery model adjacent to the calcification under the rated pressure of 12 atm when employing undersized balloons was smaller than that when only one blade faced the calcification and when lesion-identical balloon diameters were used under a nominal pressure of 6 atm. Our study suggested that undersized balloons of diameters 0.25 or 0.5 mm less than the reference diameter might be effective in not only expanding the calcified lesion but also reducing the risk of dissection.

## Introduction

Coronary artery calcification has been recognized as a risk factor associated with symptomatic coronary artery disease and major adverse cardiovascular events [1]. Conventional balloon angioplasty has a lower procedural success rate in calcified artery lesions than those in non-calcified artery lesions and often requires high dilation pressure to fracture the calcium, increasing the risk of complications [2, 3]. Calcified artery lesions are prone to result in stent under-expansion, increasing the subsequent risks of in-stent restenosis and stent thrombosis [4, 5]. Therefore, plaque modification for calcified artery lesions before stent implantation is a key procedure in percutaneous coronary intervention [6].

Cutting balloon angioplasty was introduced as a useful alternative treatment for calcified artery lesions, prior to stent implantation. The cutting-balloon catheter has three or four microsurgical metal blades mounted longitudinally on the outer surface of the balloon, which can create incisions in the calcification during dilation. In a clinical study with 92 calcified coronary artery lesions, cutting balloons appeared to be more effective than conventional balloons in acquiring acute luminal gain [7]. A study showed that the cutting balloon reduced an incidence of target vessel revascularization at 6 months in comparison with the conventional balloon in 64 coronary lesions [8] However, some studies showed that the larger lumen gain of cutting balloon angioplasty in calcified lesions is associated with the incidence of dissection, a tear formed in the artery lesion [9, 10]. A multicenter, randomized clinical trial with 1,238 patients reported that five coronary perforations occurred in the cutting balloon angioplasty group, and none occurred in the conventional angioplasty group [11]. These studies highlight that reduction of perforation of the coronary artery is crucial for the cutting balloon.

A clinical study using conventional balloon angioplasty reported that fracture of calcification was observed in 2.0% (4 of 198) of the lesions where the maximum calcium angles were ≤ 180° in the circumferential-wise direction by the cross-sectional images of optical coherence tomography [12]. This data highlights the importance of gaining more knowledge on improving clinical outcomes for calcifications whose maximum calcium angles are ≤ 180°.

A balloon-to-artery ratio of 1:1 is used for the cutting balloon, in accordance with the conventional balloon angioplasty [13]. We hypothesize that the lesion/device diameter ratio and the number of blades facing a calcified lesion may be crucial for expanding calcifications whose maximum angles are ≤ 180° adequately while avoiding dissection and vessel injuries. Therefore, in this study, we focused on 180° calcifications and finite element analyses of the expansions of cutting balloons, with different diameters under two conditions where either one or two blades face the 180° calcification models, are performed on a calcified artery model to gain mechanistic insights into calcification incisions using cutting balloons and to provide suggestions for improving the clinical outcomes of cutting balloon angioplasty.

## Materials and methods

### Geometries, mesh models, and materials for blade, cast pad, balloon, shaft, and calcified artery

**Fig 1:**
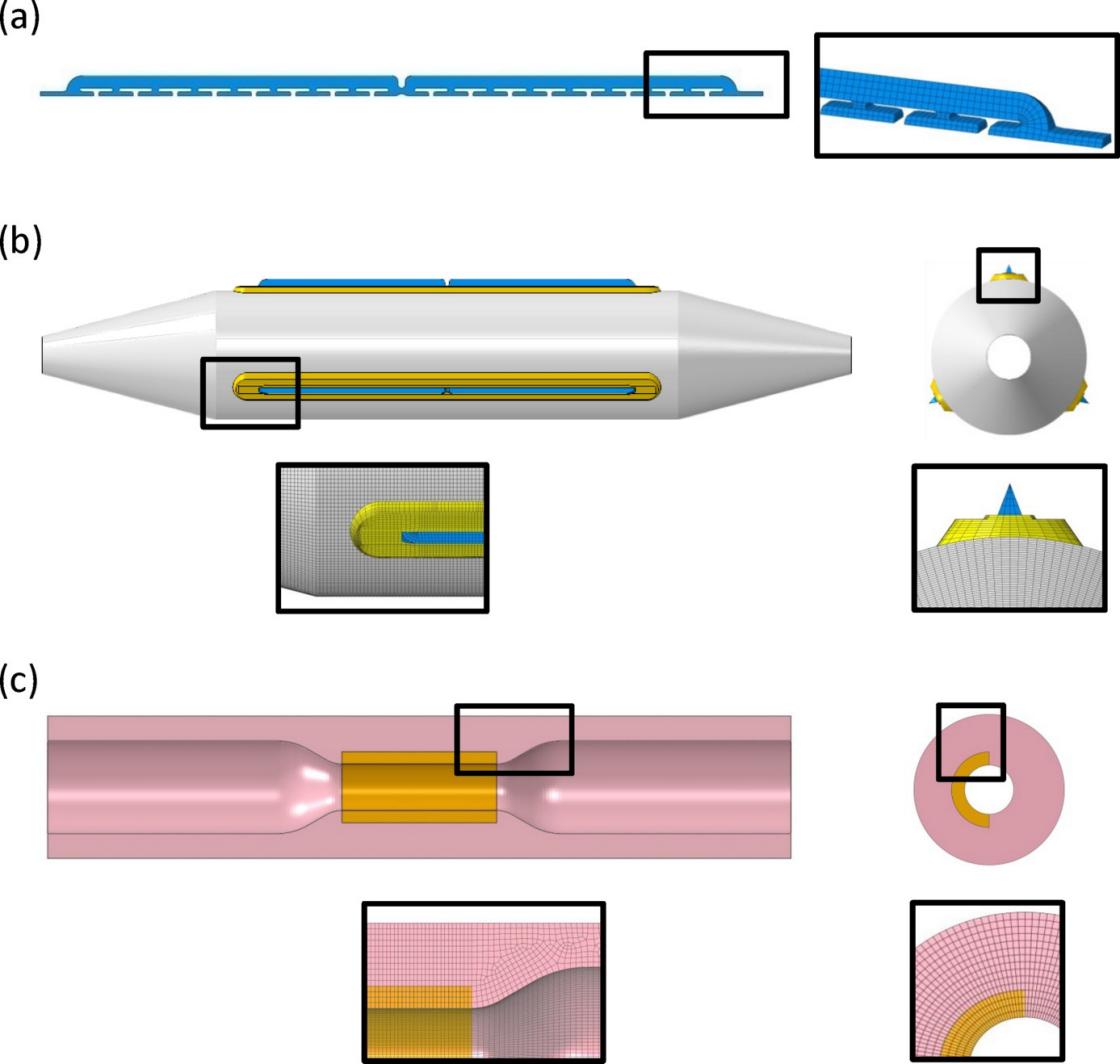
**Geometries and mesh models for (a) blade, (b) balloon with pad and blade, and (c) coronary artery with calcified artery model**.

In this study, we selected a new-generation cutting balloon, WOLVERINE ^TM^ (Boston Scientific Corporation), with balloon diameters ranging from 2 to 3 mm (2, 2.25, 2.5, 2.75, and 3 mm) and length of 10 mm. The nominal and rated inflation pressures of the cutting balloons are 6 atm and 12 atm, respectively. The cutting balloon had three microsurgical metal blades arranged lengthwise, which were bonded radially onto the balloon surface by cast pads. The blades were protected inside the folds of the wrapped balloon and would be exposed and pushed out during inflation, creating incisions and cracking the calcified lesion. The blades had a length of 8.8 mm, height of 0.25 mm, and width of 0.18 mm (Fig 1A).

The blade model was discretized with 6,256 hexahedral elements (ABAQUS element type: C3D8R), and its mechanical properties were described by an elastic-plastic model with the isotropic hardening of 316L stainless steel; i.e., Young’s modulus of 193 GPa, Poisson ratio of 0.3, yield stress of 366 MPa, and ultimate tensile strength of 675 MPa [14].

The cast pad was modeled with length 9.2 mm, height 0.1 mm, and width 0.58 mm (Fig 1B). A total of 32,376 hexahedral elements (ABAQUS element type: C3D8R) were used to mesh the geometry, and an elastic material model with Young’s modulus of 441 MPa and Poisson ratio of 0.3 was adopted.

The modelling of balloon expansion has been investigated in previous studies [15–17]. Although the actual expansion of the balloon is characterized by anisotropic and hyperelastic behavior, a linear isotropic constitutive model can be used to mimic the response of balloon expansion adequately, by deriving the initial diameter and Young’s modulus from the pressure/diameter relationship of the product, as described by De Beule et al. [18] and Ragkousis et al. [19]. In this study, the initial diameters at the balloon inflation pressure of 0 atm extrapolated from the pressure/diameter relationship of the product, and Young’s moduli for balloon sizes ranging from 2 to 3 mm were calculated using finite element analysis, as listed in Table 1. The diameters of cutting balloon calculated as a function of the balloon inflation pressure were consistent with the pressure/diameter relationship of the product within a deviation of 2% (S1 Table). A Poisson ratio of 0.4 was used. The balloon model was meshed with approximately 100,000 four-node quadrilateral membrane elements (ABAQUS element type: M3D4R) with uniform wall thickness of 0.02 mm. A rigid cylindrical surface with diameter 0.8 mm was modeled as an inner shaft inside the balloon model.

**Table 1:** Initial diameters and Young’s moduli of the balloons, obtained using finite element analysis.

Balloon diameter (mm)	2	2.25	2.5	2.75	3
Initial diameter (mm)	1.83	2.05	2.28	2.52	2.79
Young’s modulus (MPa)	337.88	364.86	418.54	446.75	533.73

A straight model of the coronary artery with length 24 mm, thickness 0.8 mm, and inner diameter 3 mm was constructed, with moderate stenosis (50% diameter stenosis) at the middle of its length. Intravascular ultrasound examinations showed that the mean length of calcification was 3.5 ± 3.7 mm, and the length with ≤ 5 mm was most frequently detected in patients [20]. Clinically, calcifications with arc ≤ 180° are difficult to be fractured by conventional balloon angioplasty [12]. Some reports have been published on the thickness of calcification. One report showed that the median thickness of calcium fracture was 450 μm when cutting balloon angioplasty or rotational atherectomy were used [21]. Another report showed that the minimum threshold of calcium thickness for fracturing was 0.24 mm when only conventional balloon angioplasty was used [12].

Therefore, in this study, a calcification model with an angle of 180°, length 5 mm, and thickness 0.4 mm was modeled and constrained by tying the adjacent two surfaces of calcification and artery models together (tie constraint). The artery model and calcification model were meshed using an eight-node hexahedral element (ABAQUS element type: C3D8R) with approximately 402,000 and 20,400 elements, respectively (Fig 1C). The mechanical behavior of the artery model was described using a first-order Mooney–Rivlin isotropic hyperelastic material model and the strain energy density function was expressed as

$$W=a_{10}\left(I_{1}-3\right)+a_{01}\left(I_{2}-3\right)$$ (1)

where *I*_1_ and *I*_2_ are the first and second invariants of the Cauchy–Green tensor and *a*_10_ and *a*_01_ are the hyperelastic constants of 18.90 kPa and 2.75 kPa referenced from the experimental data of human femoral artery [22]. A nanoindentation study showed that the elastic modulus of human calcified plaques collected from the superficial femoral artery wall was 20.1 GPa [23]. Another nanoindentation study of human carotid bifurcation plaque specimens showed that the majority of the modulus were in the range of 100 MPa to 10 GPa, with some as high as 21 GPa [24]. They reported that variations in tissue composition, incomplete mineralization, and sample preparation might be the source of the variations. Their follow-up study with reducing the cause of these potential errors showed that the modulus of calcification ranged from 15 to 25 GPa [25]. In this study, the material property of the calcification model was defined as an isotropic elastic material model with Young’s modulus of 20.1 GPa, and Poisson ratio was assumed to be 0.3. Mesh sensitivity tests were conducted to ensure the mesh convergence for each model. Mesh densities for the cutting balloon models and calcified artery model showed negligible divergence (i.e., accepting difference of less than 1% in maximum radial displacement) in comparison with finer meshes.

### Modelling of three-fold cutting balloon

**Fig 2:**
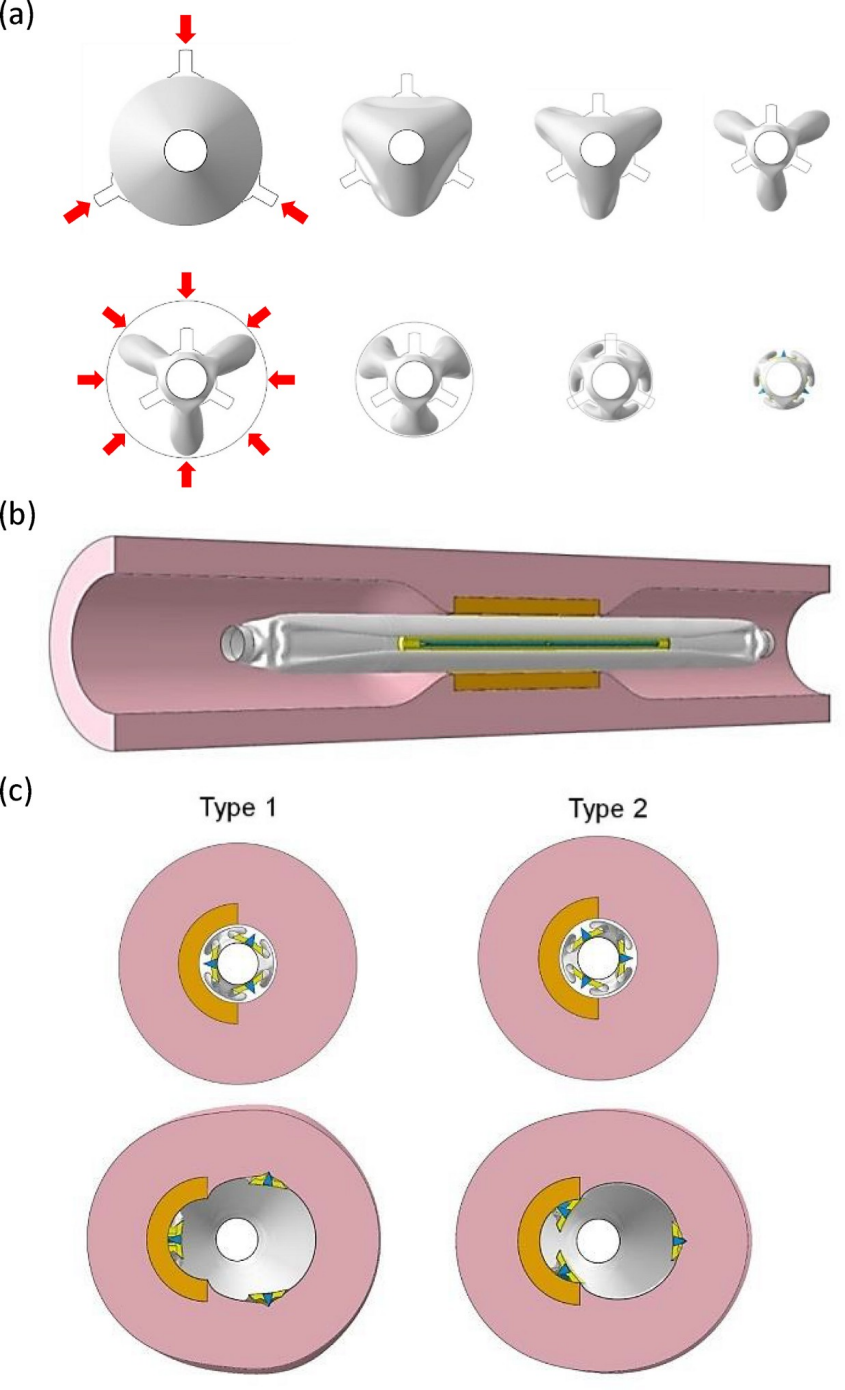
Expansion of cutting balloon in the stenotic calcified artery models. (a) Crimp and compression process of a folded balloon. (b) Configuration of cutting balloon in the calcified artery model. (c) Two types of models for the analysis (Type 1: one blade is placed inside the calcification model, Type 2: two blades are placed inside the calcification model).

A balloon-folding process was conducted to obtain a three-fold shape. First, three rigid surface models were arranged radially outside the balloon model, as crimpers, and moved toward the center axis. Then, the balloon model was crimped into a three-leaflet shape by contacting with the rigid surface models. Next, a cylindrical surface model was used to compress the three-leaflet–shaped balloon model by decreasing its diameter to 1.45 mm. Finally, three blades and cast pads were attached onto the three-fold balloon model using tie constraints to complete the cutting balloon model before expansion. To avoid numerical instability, a shell element of type S4R was assigned to the balloon model and the longitudinal ends of the balloon and shaft models were fixed in this process (Fig 2A).

### Modelling of cutting-balloon expansion

Expansion analyses of cutting balloons of different sizes were performed in the calcified artery models (Fig 2B). Pressure, up to the rated pressure of 1.216 MPa (12 atm), was applied to the inner surface of the balloon model. The shaft model, longitudinal ends of the artery model, and longitudinal ends of the balloon model were fixed. In terms of the directions of the three blades in the 180° calcified artery model, two cases were considered in the expansion simulations: Type 1 had one blade facing the calcification model while the other two blades faced the artery wall and Type 2 had two blades facing the calcification while the other faced the artery wall (Fig 2C).

Finite element analyses were performed using an Abaqus/Explicit solver (Dassault Systèmes K.K.). Each analysis was carried out as a quasi-static analysis in which the kinetic energy remained below 5% of the internal energy for each deforming material for most of the simulation. The general contact algorithm available in Abaqus/Explicit was used to define the surface-to-surface contact conditions. Contact surfaces were specified with a friction coefficient of 0.2 between the different models: balloon-artery, balloon-calcification, blade-artery, blade-calcification, cast pad-artery, cast pad-calcification, and balloon-balloon (self-contact between balloon folds).

## Results

### Stresses in the calcification models

Three principal stress components (minimum, intermediate, and maximum) with normal vectors (tensile and compression) were calculated at each nodal point of the deformed part in the finite element analysis. A clinical study suggested that a tensile stress concentration might lead to plaque fracture and dissection at the junctions between the plaque and vessel wall when a balloon was inflated within a lesion [2]. Biomechanical studies reported that the maximum principal stresses were associated with plaque rupture [26, 27]. Therefore, the maximum principal tensile stress was used to describe the stress levels in the models of the calcification and artery adjacent to the calcification.

**Fig 3:**
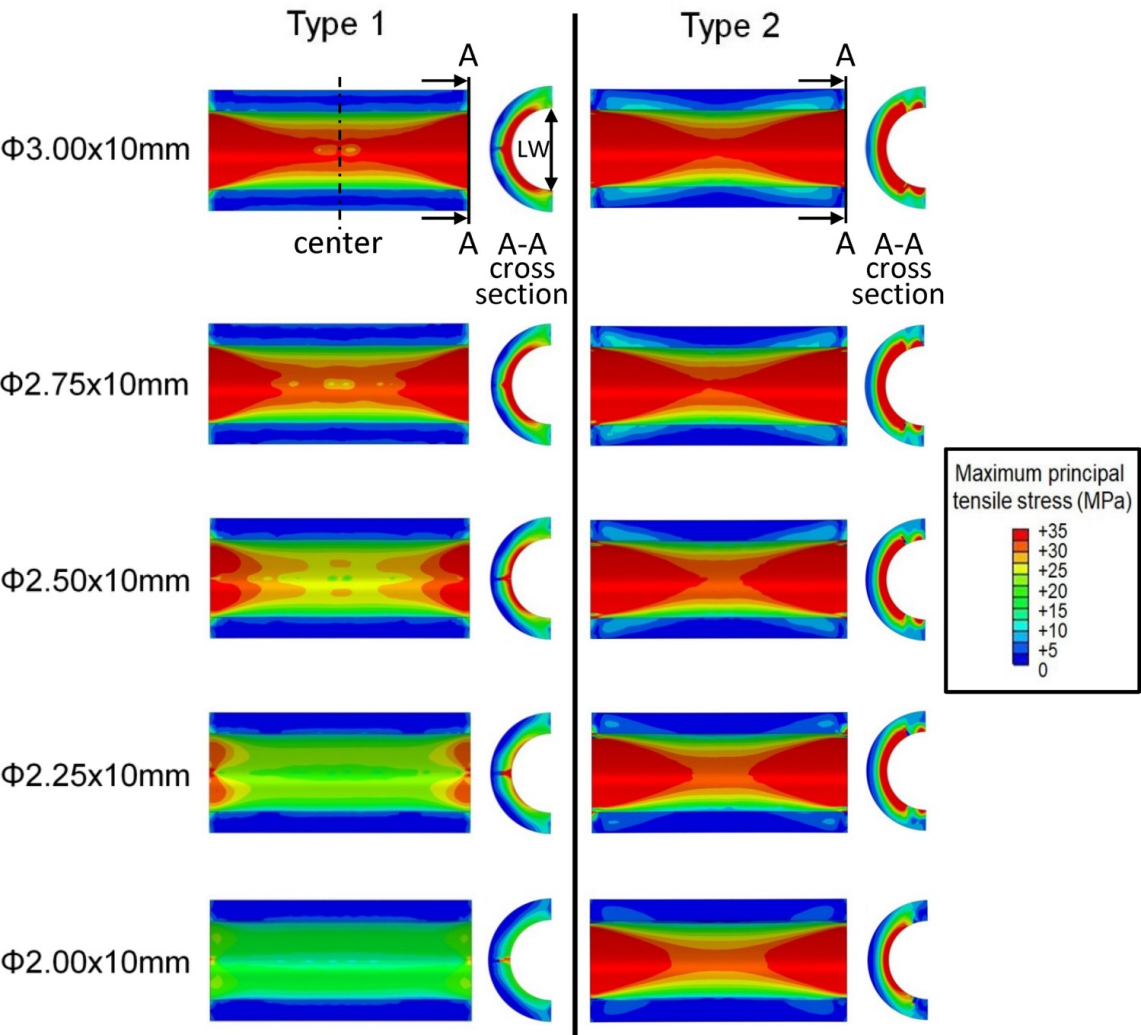
Stress distributions in the calcified artery models for Type 1 and Type 2 at rated pressure (12 atm) with five different balloon diameters. The cross-sectional stress distributions in the thickness-wise direction were at the longitudinal end of the calcification models. The location of label “A-A” is the longitudinal end of the calcification. The label “LW” denotes the lumen width of calcification at the longitudinal end at balloon inflation.

**Fig 4:**
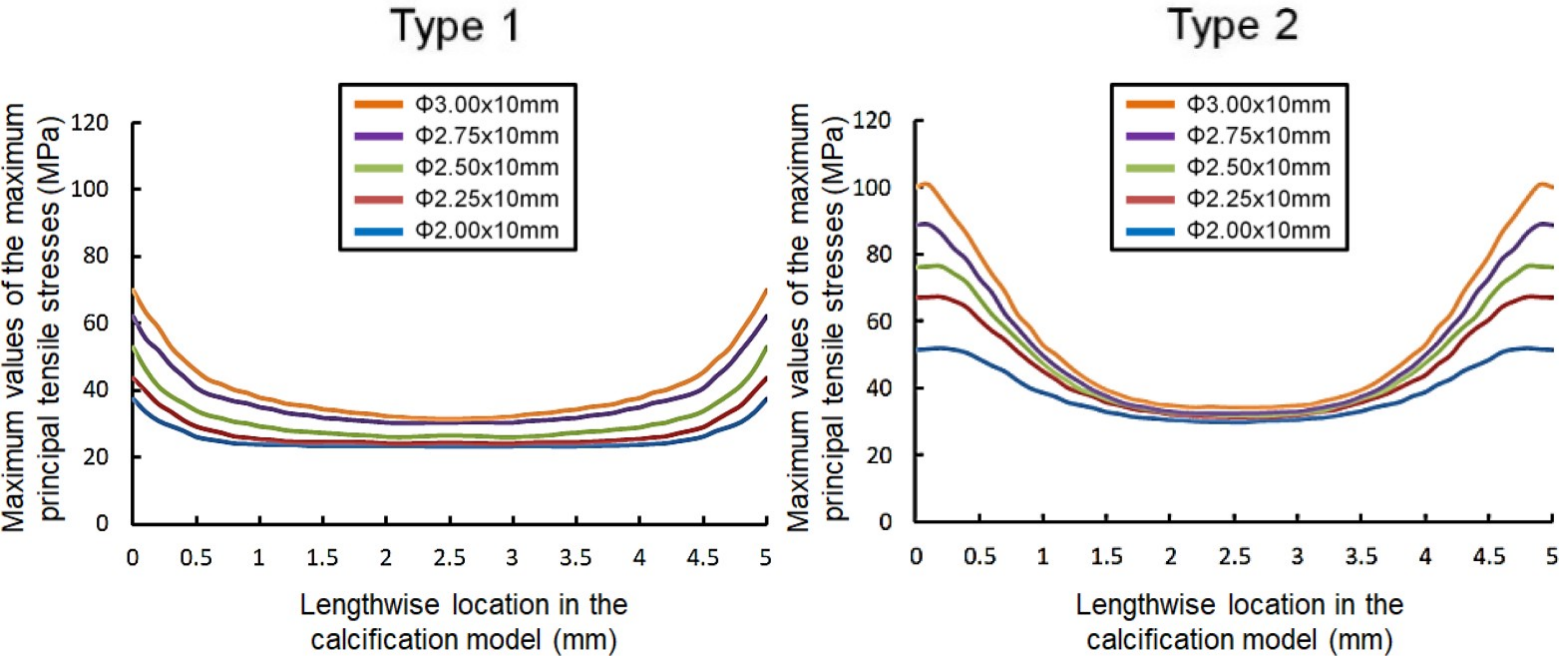
Peak values of the maximum principal tensile stresses in the calcification models along the longitudinal direction when the cutting balloon is inflated at the rated pressure (12 atm).

Fig 3 shows the contour plot of the maximum principal tensile stress. Fig 4 illustrates the peak values extracted along the lengths of the calcification models for Type 1 and Type 2 cases of cutting balloon expansion with different diameters at the rated pressure of 12 atm. It was observed that the stresses were higher at both longitudinal ends than at the center, in each calcification model. In Type 1, the stresses at the longitudinal ends of the calcifications were higher by 61, 79, 100, 105, and 123% than those at the centers of the calcification models for cutting balloon diameters of 2, 2.25, 2.5, 2.75, and 3 mm, respectively. Similarly, in Type 2, the stresses at the longitudinal ends of the calcifications were higher by 72, 114, 138, 174, and 193% than those at the centers, respectively. This was caused by the “dogbone” effect during the expansion of the balloon. Since it was reported that high maximum principal tensile stresses were associated with plaque rupture [27], these findings indicated that cracks might occur at the longitudinal ends of the calcification, first, during cutting balloon expansion.

**Fig 5:**
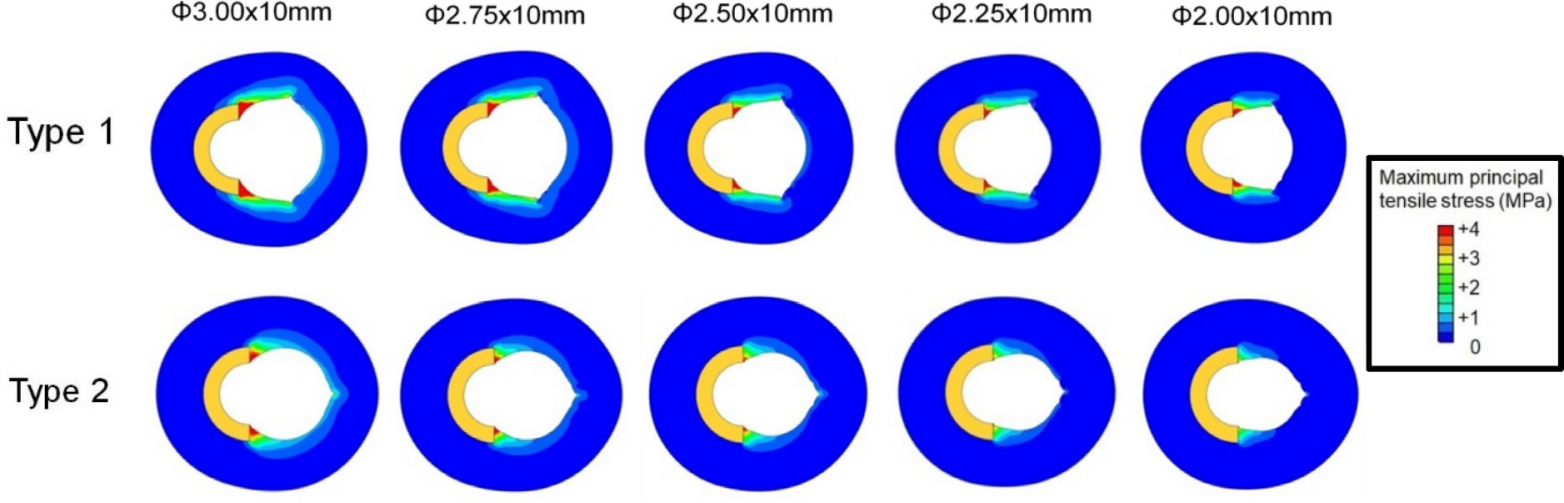
Stress distributions in the artery model for Type 1 and Type 2 at the rated pressure (12 atm) for five different balloon diameters.

**Fig 6:**
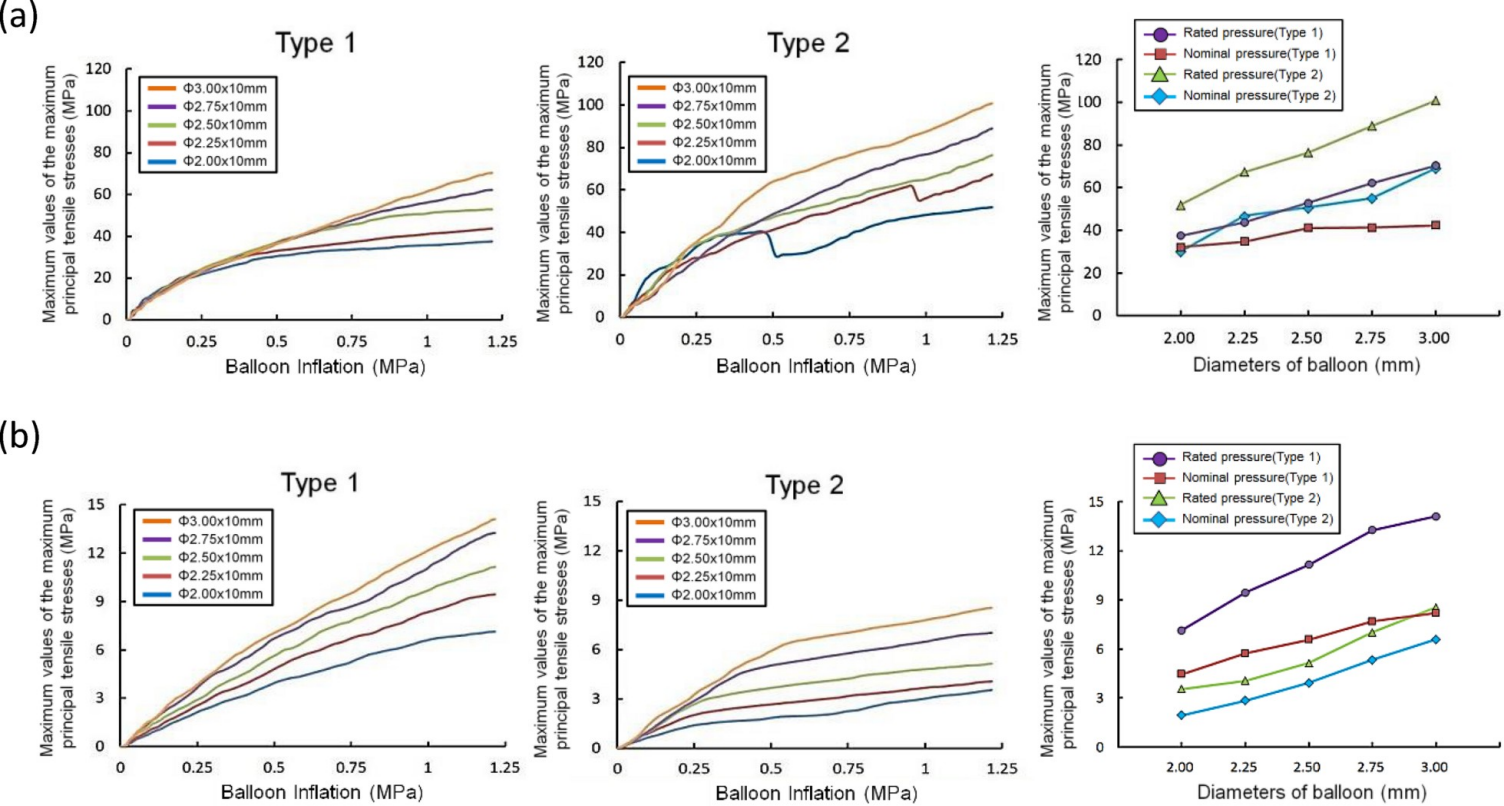
Effects of cutting balloon diameter and blade direction on the maximum principal tensile stresses in the models of the calcification and artery adjacent to the calcification. (a) Maximum principal tensile stresses in the calcification model for Type 1 and Type 2 for five different balloon diameters. (b) Maximum principal tensile stresses in the artery model adjacent to the calcification for Type 1 and Type 2 for five different balloon diameters.

Fig 5 shows the maximum principal tensile stresses generated in the coronary artery model adjacent to the calcification for Types 1 and 2, expanded with different balloon diameters at 12 atm. All the cross-sections were drawn at the longitudinal end positions of the artery models connected with the calcification models where the peak values occurred. In Fig 6A, the peak values of the maximum principal tensile stresses in the calcification models for Types 1 and 2, from pressures of 0 to 12 atm, are extracted. All peak values for Type 1 and Type 2 models, at both nominal and rated pressures, are listed in Table 2. Larger balloon diameters resulted in higher levels of stress for both the types, except for the cutting balloon diameter of 2 mm at nominal pressure in the Type 2 model (Table 2). Particularly, in Type 2, the peak values for 2 and 2.25 mm diameters were observed to creep down rapidly, near the pressures of 0.5 and 0.9 MPa, respectively. The results indicated that the calcification expansion ability of the cutting balloon in the case of Type 1 under the rated pressure of 12 atm was comparable to that in the case of Type 2 under the nominal pressure of 6 atm (Fig 6A). Moreover, in the case of Type 2, under both the nominal and rated pressures, larger stress concentrations occurred in the calcification models even using undersized balloons of diameter 2.5 and 2.75 mm, in comparison with the case of Type 1 using a balloon of diameter 3 mm (Table 2).

**Table 2:** Peak values of the maximum principal tensile stresses in the calcification models.

Balloon diameter (mm)		2	2.25	2.5	2.75	3
Peak values of the maximum principal tensile stresses (MPa)						
Nominal pressure (6 atm)	Type 1	32.2	34.8	41.1	41.3	42.4
	Type 2	30.2	46.7	50.7	55.1	69.1
Rated pressure (12 atm)	Type 1	37.5	43.7	52.9	62.2	70.4
	Type 2	51.9	67.3	76.4	88.9	100.8

The lumen width corresponding to the inner distance of calcification during expansion at the longitudinal and circumferential ends (denoted as LW in Fig 3) were calculated and listed in Table 3. The lumen width in the case of Type 2 increased in comparison with that in the case of Type 1 for each balloon diameter at both the nominal and rated pressures.

**Table 3:** Lumen width at the inner surface of the calcification model at the longitudinal and circumferential ends.

Balloon diameter (mm)		2	2.25	2.5	2.75	3
Lumen width (mm)						
Nominal pressure (6 atm)	Type 1	1.504	1.506	1.507	1.509	1.513
	Type 2	1.509	1.511	1.515	1.517	1.519
Rated pressure (12 atm)	Type 1	1.508	1.513	1.517	1.520	1.524
	Type 2	1.518	1.528	1.536	1.543	1.552

### Stresses in the artery models

Fig 6B shows the peak values of the maximum principal tensile stresses generated in the artery models adjacent to the calcification under pressures ranging from 0 to 12 atm. The peak values at the nominal and rated pressures for Type 1 and Type 2 are listed in Table 4. Larger balloon diameters resulted in higher levels of stress for both the types in the artery models adjacent to the calcification. The peak values at both nominal and rated pressures for Type 1 were higher than those for Type 2. Interestingly, the maximum principal stress calculated in the artery adjacent to the calcification in the case of Type 2 under even the rated balloon inflation pressure of 12 atm with the undersized balloons of 2.75 mm was smaller than that in the case of Type 1 under the nominal balloon inflation pressure of 6 atm with the 3.0 mm reference diameter balloon (Table 3 and Fig 6B). These findings suggest that in the case of Type 2, the risk of dissection or perforation upon expanding the balloon with the rated pressure can be reduced, in comparison with the balloon expansion under nominal pressure in Type 1, when at least 0.25 mm undersized balloons are employed in comparison with the reference diameter.

**Table 4:** Peak values of the maximum principal tensile stresses in the model of artery adjacent to the calcification.

Balloon diameter (mm)		2	2.25	2.5	2.75	3
Peak values of the maximum principal tensile stresses (MPa)						
Nominal pressure (6 atm)	Type 1	4.45	5.73	6.57	7.69	8.19
	Type 2	1.96	2.85	3.92	5.34	6.59
Rated pressure (12 atm)	Type 1	7.15	9.46	11.16	13.27	14.12
	Type 2	3.55	4.06	5.15	7.02	8.54

### Effects of balloon diameter and blade direction

**Fig 7:**
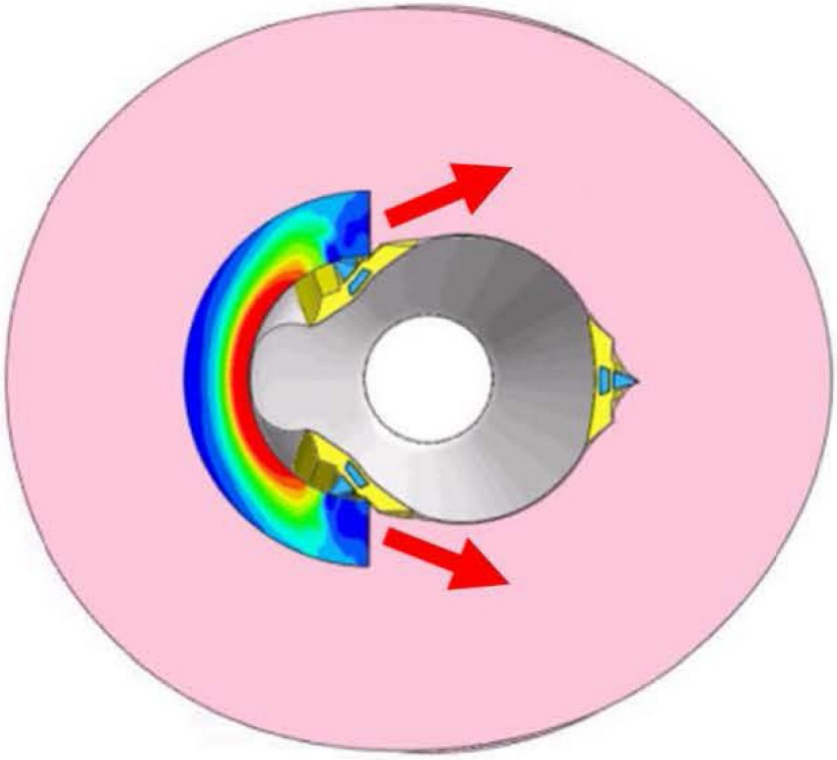
Incidence of slippage during the inflation of balloons of diameters 2 and 2.25 mm.

In Fig 6A, a reduction in stress is observed during the expansion of the 2- and 2.25-mm-diameter cutting balloons of Type 2 near the pressures of 0.5 and 0.9 MPa, respectively. This resulted in lower peak stresses in Type 2, compared to that in Type 1 at nominal pressure. An obvious slippage of the blades was observed and two of the blades were dragged in opposite directions during the expansion (the red arrows in Fig 7).

**Fig 8:**
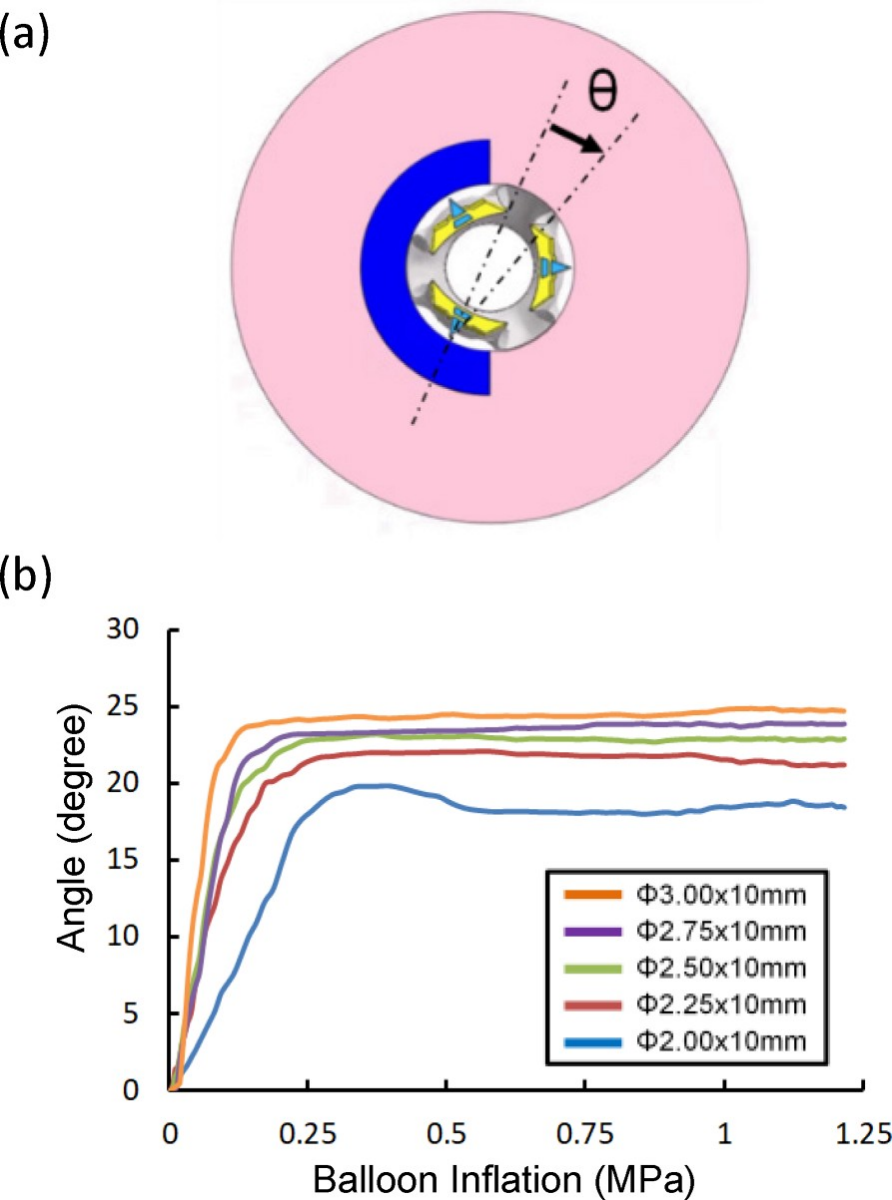
Changes in the angles of the blades during inflation. (a) Change in the angle of the blade facing the calcification model during expansion. (b) Comparison of angle changes among 2, 2.25, 2.5, 2.75, and 3 mm cutting balloons for the entire pressure range.

Moreover, in Type 2 expansion, the stresses for the 2-mm-diameter cutting balloon expansion were higher than those for the other balloon diameters, from the incipient pressure of 0.06 MPa to 0.15 MPa (Fig 6A). The change in the angle of the blade facing the calcification model was measured from the center line of the blade cross-section during expansion (Fig 8A). In Fig 8B, the angle change for the 2-mm-diameter cutting balloon was obviously lower than that for the 2.25-, 2.5-, 2.75-, and 3-mm-diameter cutting balloons for the entire pressure range including 0.06 to 0.15 MPa. Therefore, a higher acting force was generated in the normal direction of the calcification model as to the 2-mm-diamter cutting balloon for Type 2.

## Discussion

We performed the finite element analysis of expansion simulations of the cutting balloons with five different balloon diameters in the 180° calcified artery model in two cases. Type 1 simulated the case that one blade faced the calcification while the other two blades faced the artery wall. Type 2 simulated the case that two blades faced the calcification while the other faced the artery wall. The calcification expansion ability was distinctly higher when two blades faced the calcification (Type 2) than when one blade faced the calcification (Type 1). Moreover, when two blades faced the calcification (Type 2) with undersized balloons of diameters 0.25 or 0. 5 mm less than the reference diameter, larger stresses were calculated in the calcification in comparison to simulations where one blade faced the calcification (Type 1) with a balloon with the reference diameter. In terms of the stress concentration in the artery adjacent to the calcification, the peak values were higher when one blade faced the calcification (Type 1) than when two blades faced the calcification (Type 2). Interestingly, the maximum principal stress calculated in the artery adjacent to the calcification in the case where two blades faced the calcification (Type 2) under even the rated balloon inflation pressure of 12 atm (higher by 6 atm in comparison with the nominal pressure of 6 atm) with the undersized 2.75-mm-diameter balloon was smaller than that in the case where one blade faced the calcification (Type 1) under the nominal balloon inflation pressure of 6 atm with the 3.0-mm reference-diameter balloon. These findings suggest that in the case where two blades face the calcification, undersized balloons of diameters 0.25 or 0.5 mm less than the reference diameter might be effective for not only expanding the calcified lesion but also reducing the risk of dissection and perforation.

Cutting balloon angioplasty provided adequate lumen expansion in the calcified lesions, but suffered from the possibility of arterial dissection [9]. In our simulation study, a larger balloon diameter resulted in a higher level of stress in both the calcification and artery adjacent to the calcification, which might lead to plaque rupture and arterial dissection. However, a comparison of the two types of expansions in 180° calcified artery models indicated that the arrangement where two blades faced the calcification as opposed to one blade could generate higher stresses in the calcification for potentially inducing fracture, while generating lower stresses in the artery adjacent to the calcification, so as to be less traumatic to the arterial wall.

In a clinical setting, there are no measures to guide two blades facing the calcification. However, we have shown that three-time repetition of delivery-balloon inflation for stent deployment increased the luminal cross-sectional area [28, 29]. Repeating the cutting balloon inflation by changing the rotation of the cutting balloon catheter may change the direction of the blades and may be able to adjust two blades toward the calcification.

There are some limitations in this work. First, the calcified artery was simply characterized using the parameters of thickness and arc of 180° for the calcification, although the thickness and degree of calcification are clinically relevant. There are also some simplifications in the artery. The arterial vessel layers (intima, media and adventitia) and three types of plaque material (cellular, hypocellular and calcified) have been modeled to describe realistic coronary arteries [30, 31]. However, because the focus of this simulation was to investigate the effects of the lesion/device diameter ratio and the number of blades facing a calcification, the use of a simplified artery was considered acceptable. Second, our modelling approach simulated indenting the calcification rather than cutting or breaking the calcification. The fracture behaviors of the calcification model and the dissection of the calcification and artery wall were not considered in this work. Cracks in the calcification model and separation of the calcification and artery may occur at the regions with high-level stresses generated by the expansion of the cutting balloons. Based on the findings in this simulation, we are planning to perform an experimental investigation using a 180° calcified artery replica to verify the effects of vessel/device diameter ratio and the number of blades facing the calcification. Third, in the present study, inertia effect such as high-speed impact by cutting balloon dilation on the calcified artery model was neglected.

Nevertheless, our study suggested that when non-circular calcified lesion is treated using the cutting balloon, adjustment of two blades to the calcification and use of undersized balloons of diameters 0.25 mm or 0.5 mm less than the reference diameter might be effective in not only expanding the calcified lesion but also reducing the risk of dissection and vessel perforation.

## Supporting information

S1 Table Pressure/diameter relationship for each cutting balloon obtained from finite element analysis and the manufacturer. (PDF)
