# Supplementary material for: Finite element analysis of cutting balloon expansion in a calcified artery model of circular angle 180°: Effects of balloon-to-diameter ratio and number of blades facing calcification on potential calcification fracturing and perforation reduction
Source: PLoS One. 2021 May 13;16(5):e0251404. doi: 10.1371/journal.pone.0251404 (PMC8118280; doi:10.1371/journal.pone.0251404)
Supplement: S1 Table — (PDF) [file pone.0251404.s001.pdf]

Pressure		Balloon diameter (mm)									
		Finite element analysis					Product data provide by the manufacturer				
atm	MPa	2	2.25	2.5	2.75	3	2	2.25	2.5	2.75	3
3	0.304	1.89	2.13	2.37	2.61	2.88	1.90	2.14	2.38	2.62	2.88
4	0.405	1.92	2.16	2.40	2.65	2.92	1.95	2.18	2.43	2.69	2.94
5	0.507	1.95	2.19	2.44	2.69	2.96	1.98	2.22	2.48	2.73	2.99
6	0.608	1.98	2.23	2.47	2.73	3.00	2.02	2.26	2.52	2.78	3.06
7	0.709	2.01	2.26	2.51	2.78	3.05	2.05	2.30	2.56	2.83	3.10
8	0.811	2.05	2.30	2.56	2.83	3.10	2.08	2.33	2.60	2.88	3.15
9	0.912	2.08	2.34	2.60	2.88	3.15	2.11	2.36	2.64	2.91	3.18
10	1.013	2.12	2.39	2.65	2.94	3.20	2.13	2.39	2.67	2.95	3.22
11	1.115	2.17	2.44	2.70	2.99	3.26	2.15	2.41	2.69	2.97	3.25
12	1.216	2.21	2.49	2.75	3.06	3.32	2.17	2.44	2.71	3.00	3.28
